# Wulingsan Alleviates MAFLD by Activating Autophagy via Regulating the AMPK/mTOR/ULK1 Signaling Pathway

**DOI:** 10.1155/2024/9777866

**Published:** 2024-07-13

**Authors:** Yaning Biao, Dantong Li, Yixin Zhang, Jingmiao Gao, Yi Xiao, Zehe Yu, Li Li

**Affiliations:** ^1^ School of Basic Medicine Hebei University of Chinese Medicine, Shijiazhuang, Hebei, China; ^2^ School of Pharmacy Hebei University of Chinese Medicine, Shijiazhuang, Hebei, China; ^3^ School of Pharmacy Hebei Medical University, Shijiazhuang, Hebei, China

## Abstract

Here, we presented the study of the molecular mechanisms underlying the action of Wulingsan (WLS) in rats with metabolic-associated fatty liver disease (MAFLD) induced by a high-fat diet (HFD). High-performance liquid chromatography was employed to identify the chemical components of WLS. After 2 weeks of HFD induction, MAFLD rats were treated with WLS in three different doses for 6 weeks, a positive control treatment or with a vehicle. Lipid metabolism, liver function, oxidative stress, and inflammatory factors as well as pathomorphological changes in liver parenchyma were assessed in all groups. Finally, the expressions of autophagy-related markers, adenosine monophosphate-activated protein kinase (AMPK)/mechanistic target of rapamycin (mTOR)/unc-51-like kinase-1 (ULK1) signaling pathway-related genes, and proteins in liver were detected. The results revealed that WLS significantly ameliorated liver injury, the dysfunction of the lipid metabolism, the oxidative stress, and overall inflammatory status. Furthermore, WLS increased the expressions of LC3B-II, Beclin1, p-AMPK, and ULK1, along with decreased p62, p-mTOR, and sterol regulatory element-binding protein-1c levels. In conclusion, we showed that WLS is capable of alleviating HFD-induced MAFLD by improving lipid accumulation, suppressing oxidative stress and inflammation, and promoting autophagy.

## 1. Introduction

Nonalcoholic fatty liver disease (NAFLD) has become the leading cause of chronic liver disease, and it was recently defined as metabolic-associated fatty liver disease (MAFLD) to emphasize the role of metabolic dysfunction in this condition [1]. MAFLD manifests as hepatic steatosis in combination with overweight/obesity, type 2 diabetes, or metabolic dysregulation. It affects approximately 25.24% adults worldwide, with the highest prevalence rates in the Middle East and South America [2, 3]. Moreover, the prevalence of MAFLD also increases among children and young adolescents [4]. MAFLD can progress to nonalcoholic steatohepatitis (NASH), leading to hepatic fibrosis and hepatocellular carcinoma [5]. Furthermore, MAFLD is associated with an increased risk of type 2 diabetes, cardiovascular disorders, and certain cancers, further exacerbating the health and economic burden on society [6, 7]. However, the available pharmacological therapies for this disease remain limited.

Although the development of MAFLD is a complex and multifactorial process, the classical “two-hit” theory suggested that oxidative stress and inflammation play crucial roles in its pathogenesis. The “first hit” is characterized by hepatic lipid accumulation and insulin resistance. Subsequently, the “second hit” emerges, involving inflammatory factors, mitochondrial dysfunction, and oxidative stress, which further exacerbate MAFLD and lead to NASH. Recent studies have increasingly suggested the relevance of autophagy in delaying the progression of MAFLD [8]. Autophagy, a lysosomal degradation pathway, reduces hepatic lipid deposition, improves insulin resistance, and inhibits oxidative injury and inflammation [9]. Lipid metabolism disorder is a key link in the pathogenesis of MAFLD, and autophagy plays a crucial role in regulating hepatic lipid metabolism [10, 11]. Lipophagy, a selective autophagic process for the catabolism of lipid droplets in the lysosomes, supplies free fatty acid (FFA) to produce energy and ketone bodies in hepatocytes through *β*-oxidation [12]. Research has shown that activation of autophagy could significantly inhibit hepatic steatosis, while defective autophagy may lead to hepatic steatosis and further accelerate the development and progression of MAFLD [13]. Adenosine monophosphate-activated protein kinase (AMPK), an essential energy regulator, promotes autophagy by inhibiting the mechanistic target of rapamycin (mTOR) and activating unc-51-like kinase-1 (ULK1) [14]. Additionally, phosphorylated AMPK decreases the expression of sterol regulatory element-binding protein-1c (SREBP-1c), thereby reducing adipogenesis [15]. Activating AMPK/ULK1 signaling and inhibiting mTOR phosphorylation could reduce lipid accumulation in high-fat diet (HFD)-induced obese mouse [16]. However, FFA-induced HepG2 cells were treated with AMPK inhibitor Compound C (CC), or silence of ULK1 by transfection with siRNA could inhibit hepatic autophagy and promote lipid accumulation [17]. These findings demonstrated that targeting the AMPK/mTOR/ULK1 signaling pathway and promoting autophagy may offer new therapeutic strategies for MAFLD.

In China, traditional Chinese medicine (TCM) has become widespread for over 2000 years. TCM follows the principle of “multiple ingredients-multiple targets-multiple pathways” and holds promise as a novel approach for treating MAFLD. One TCM prescription, Wulingsan (WLS), has traditionally been used to regulate body fluid homeostasis. Its first mention can be traced back to the third century in the *Shanghan Lun*, written by Zhang ZhongJing. WLS is composed of zhuling (Polyporus), fuling (Poria cocos), zexie (Alismatis rhizoma), baizhu (Atractylodis macrocephalae rhizoma), and guizhi (Cinnamomi ramulus), and it is known for its efficacy in warming the *yang*, strengthening the spleen, and eliminating dampness to address phlegm-related issues. In TCM, MAFLD is often attributed to spleen dysfunction and the accumulation of “phlegm-dampness.” Although “phlegm-dampness” is an abstract concept in the TCM system, we understand its close association with body fluid homeostasis. Modern research has demonstrated that WLS could regulate lipid metabolism in obese rats [18]. Various extracts and bioactive monomers of WLS have been found to ameliorate inflammation and oxidative stress. Polyporus polysaccharide induced autophagy to inhibit the proliferation of cancer cell [19], and reduced inflammation in lung and kidney fibrosis mouse [20, 21]. Poria cocos polysaccharide protected liver injury by suppressing inflammation and oxidative stress [22]. In addition, Poria cocos extract also alleviated HFD-induced MAFLD by regulating lipid homeostasis and bile acid metabolism [23]. Alisol A 24-acetate, a triterpene from Alismatis rhizoma, could ameliorate hepatic steatosis and inhibit inflammation [24]. Atractylodis macrocephalae rhizoma could promote energy metabolism to prevent obesity [25]. Furthermore, essential oil from Atractylodis macrocephalae rhizoma has antioxidant and anti-inflammatory activities [26]. Cinnamomi ramulus essential oil showed anti-inflammatory effects in N-acylethanolamine acid amidase-overexpressing HEK293 cells [27]. However, it is unclear whether WLS could activate autophagy to suppress oxidative stress and inflammation and thus ameliorate MAFLD. Therefore, this study aimed to explore the protective effects of WLS on MAFLD rats induced by HFD and elucidate the role of WLS in regulating the AMPK/mTOR/ULK1 signaling pathway.

## 2. Materials and Methods

### 2.1. Preparation of Experimental Drugs

The formula granules of WLS were zhuling (Polyporus) 9 g, fuling (Poria cocos) 9 g, zexie (Alismatis rhizoma) 12 g, baizhu (Atractylodis macrocephalae rhizoma) 6 g, and guizhi (Cinnamomi ramulus) 9 g. Polyene phosphatidylcholine capsules (PPC, ABJD069B) were obtained from Beijing Sanofi Pharmaceutical Co., Ltd. (Beijing, China). Distilled water at 100°C was used to dissolve WLS and PPC.

### 2.2. Chemicals and Reagents

The standards for ferulic acid (AB175), cinnamic acid (AB0479), cinnamaldehyde (AB2431), alisol C-23-acetate (AB1085), atractylenolide II (AB0313), and atractylenolide III (AB0316) were obtained from Chengdu Alfa Biotechnology Co., Ltd. (purity ≥ 98%, Chengdu, China). Kits for measuring total cholesterol (TC, A111-2-1), triacylglycerol (TG, A110-2-1), alanine transaminase (ALT, A042-1-1), aspartate aminotransferase (AST, C009-2-1), high-density lipoprotein cholesterol (HDL-C, A110-2-1), low-density lipoprotein cholesterol (LDL-C, A112-1-1), FFA (A113-1-1), superoxide dismutase (SOD, A003-2-2), catalase (CAT, A006-2-1), glutathione (GSH, A006-1-1), and malondialdehyde (MDA, A007-1-1) were purchased from Nanjing Jiancheng Bioengineering Institute (Nanjing, China). Kits for interleukin (IL)-1*β* (SXR026) and IL-6 (SXR032) were obtained from Shanghai Senxiong Biomedical Technology (Shanghai, China). Antibodies against AMPK (AF6423), phosphorylated AMPK (p-AMPK, Thr172, AF3423), mTOR (AF6308), phosphorylated mTOR (p-mTOR, AF3308), light chain 3B (LC3B, AF4615), and Beclin1 (AF5128) were provided by Affinity Biosciences (Cincinnati, OH, United States). Antibodies against p62 (bs-2951R), ULK1 (bs-1926R), and SREBP-1c (bs-1402R) were obtained from Beijing Bioss Biotechnology Co., Ltd. (Beijing, China). The chow and HFD (2022011106) were provided by Beijing Keao Xieli Feed Co., Ltd. (Beijing, China).

### 2.3. High-Performance Liquid Chromatography (HPLC) Analysis

The main chemical components of WLS were analyzed using HPLC (Essentia LC-16 system, SHIMADZU, Tokyo, Japan), and chromatographic separation was conducted on a Shim-pack GIST C18 column (4.6 mm × 150 mm, 5 *μ*m). The WLS formula granules were ground into powder, and 0.2 g of the powder was sonicated in 25 mL of 75% methanol for 30 min. The resulting solution was then passed through a 0.45 *μ*m microporous filter membrane, and the filtrate was subjected to HPLC analysis. The mobile phase comprised acetonitrile (solvent A) and 0.1% phosphoric acid in water (solvent B). The flow rate was set at 1.0 mL/min. The gradient elution program was as follows: 0–20 min, 95−75% B; 20–30 min, 75−57% B; 30–50 min, 57−20% B; 50–60 min, 20−5% B. The column temperature was maintained at 35°C, and the chromatogram was monitored at 254 nm and 220 nm. The autosampler was configured to inject 10 *µ*L.

### 2.4. Animals

Sixty male Sprague-Dawley rats (weighing 180 ± 20 g) were obtained from Beijing Vital River Laboratory Animal Technology Co., Ltd. (Beijing, China) and housed in a controlled environment with a 12-h light-dark cycle, as well as regulated temperature and humidity. All animal procedures were conducted following the guidelines approved by the Ethics Committee of the Hebei University of Chinese Medicine (No.DWLL2022203126).

After 1 week of acclimatization, the rats were randomly divided into two groups: the control group (*n* = 10) and the HFD group (*n* = 50). The control group received drinking water and standard chow, while the HFD group was given a HFD (78.8% basic feed, 15% lard, 5% sucrose, 1% cholesterol, and 0.2% sodium cholate) for 8 weeks. Following 2 weeks of HFD feeding, the rats in the HFD group were further divided into five subgroups (*n* = 10): the model group received normal saline via gavage (10 mL/kg), the PPC group received PPC (0.144 g/kg) as a positive control, and the WLS groups were administered different doses of WLS (0.777, 1.554, and 3.108 g/kg, equivalent to 1, 2, and 4 times the clinically equivalent dose, referred to as L-WLS, M-WLS, and H-WLS, respectively). Each group of rats were treated by gavage at 8:30 a.m. daily. The dosage of drugs was calculated according to “Methodology on Chinese medicinal pharmacology” [28].

After 6 weeks of treating, rats were anesthetized with an intraperitoneal injection of 3% sodium pentobarbital at a dose of 1 mL/kg. Then, blood and liver were rapidly collected.

### 2.5. Biochemical Analysis

Serum lipids (TC, TG, HDL-C, and LDL-C), liver function indicators (ALT and AST), and liver lipids (TC, TG, and FFA) were determined using a biochemical analysis (Humalyzer 2000, Germany). Enzyme-linked immunosorbent assay (ELISA) was employed to assess the serum levels of IL-1*β* and IL-6. The activities or levels of SOD, CAT, GSH, and MDA in serum were measured using respective kits according to the manufacturer's instructions.

### 2.6. Liver Histopathological Evaluations

Liver tissues were fixed in 4% paraformaldehyde, embedded in paraffin, dehydrated, and sliced for hematoxylin and eosin (H&E) staining. At the same time, NAFLD activity score (NAS) was used to score the liver histomorphology [29], and the specific scoring criteria are shown in Table 1.

Additionally, 5-*μ*m frozen liver sections were stained with oil red O staining. The stained sections were observed under a microscope (Nikon Eclipse E100, Tokyo, Japan). The staining area (Area) and integral absorbance (IA) of oil red O were determined using Image Pro Plus, and IOD (sum)/Area (sum) was used as the final result.

### 2.7. Quantitative Real-Time PCR (qRT-PCR) Analysis

Total RNA was extracted from liver tissues using Trizol reagent and reverse-transcribed into cDNA using a TianScript RT reagent kit. The primer sequences are provided in Table 2. Amplification was performed under the following conditions: 95°C for 15 min, 40 cycles of 95°C for 10 s, and 58°C for 60 s. The expressions of genes were analyzed using the 2^−ΔΔCt^ method.

### 2.8. Immunohistochemistry Staining (IHC)

The expressions of p62, ULK1, and SREBP-1c in liver were assessed using IHC. Liver tissues fixed in 4% paraformaldehyde were embedded in paraffin, dehydrated, subjected to antigen retrieval, and treated with 3% H_2_O_2_ to block endogenous peroxidase activity. The tissues were then blocked with 3% BSA and incubated overnight at 4°C with the primary antibody (1 : 500), followed by incubation with the secondary antibody (1 : 500). Subsequently, the slides were stained using a DAB color developing solution and counterstained with hematoxylin. The stained slides were observed under a microscope, and the presence of brown-yellow staining was considered as positive expression. Image Pro Plus 6.0 software was used to measure the integrated optical density value (IOD) and pixel area (Area) of each image, and the ratio of IOD (sum)/Area (sum) was calculated.

### 2.9. Western Blot Analysis

The liver tissues were homogenized in 1 mL RIPA lysis buffer and 10 *μ*L PMSF for extracting protein. The extracted proteins were then separated by 10% SDS-PAGE and transferred onto a PVDF membrane. The membrane was blocked with a rapid closure solution for 5 min and incubated overnight at 4°C with specific primary antibodies (1 : 1000) against AMPK, p-AMPK, mTOR, p-mTOR, LC3B, and Beclin1. Afterward, the membrane was incubated with the secondary antibody (1 : 5000) for 30 min. Protein bands on the membrane were visualized using an enhanced chemiluminescence (ECL) reaction and detected using a multifunctional imaging system (Fusion FX5 Spectra, Vilbe, France). The expressions of proteins were analyzed using Image J.

### 2.10. Statistical Analysis

The data were presented as mean ± standard deviation (SD) and analyzed using SPSS 19.0 (IBM, Armonk, NY, USA). One-way analysis of variance (ANOVA) was performed to analyze the data, followed by a Tukey's test or Dunnett'T3 test for post hoc comparisons. *P* < 0.05 was considered statistically significant.

## 3. Results

### 3.1. Identification of Chemical Components in WLS by HPLC

**Figure 1:**
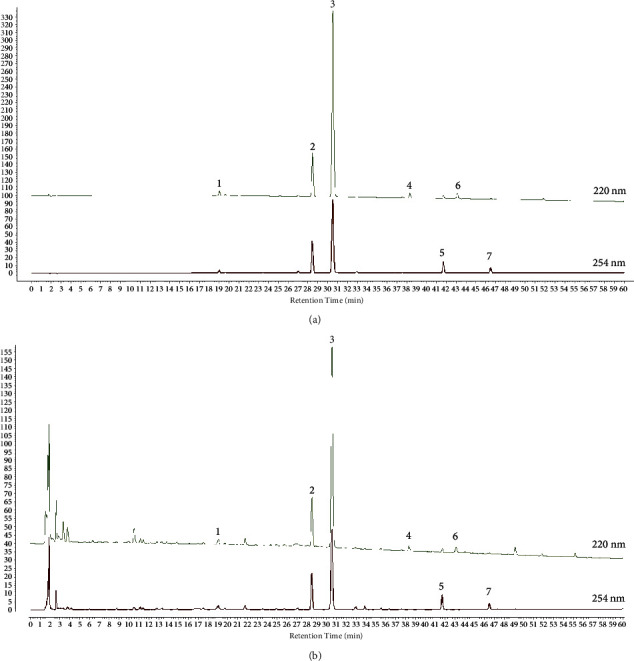
Identification of chemical compounds in WLS by HPLC. (a) Chromatogram of the mixed standard. (b) Chromatogram of WLS samples (1: ferulic acid; 2: cinnamic acid; 3: cinnamaldehyde; 4: atractylenolide III; 5: alisol C-23-acetate; 6: atractylenolide II; 7: atractylenolide I).

As shown in Figure 1, a total of 7 chemical components were determined in WLS, named ferulic acid, cinnamic acid, cinnamaldehyde, alisol C-23-acetate, atractylenolide I, atractylenolide III, and atractylenolide II. The contents of these components are presented in Table 3. To perform linear regression analysis, standard curves of peak area (Y) and concentration (X) of the standards were plotted. As indicated in Table 3, 7 components exhibited a strong linear relationship under the given chromatographic conditions.

### 3.2. WLS Attenuated Liver Injury in MAFLD Rats

**Figure 2:**
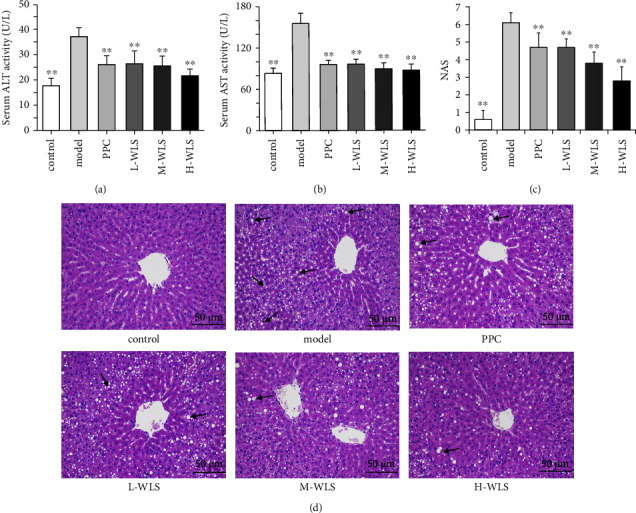
Effect of WLS on liver injury in MAFLD rats. (a, b) Serum activities of ALT and AST. (c) NAS. (d) H&E staining of liver tissue (×200). Results were expressed as the mean ± SD, *n* = 10. ^*∗∗*^*P* < 0.01, compared with the model group.

In the model group, a long-term HFD resulted in severe liver damage, leading to a significant increase in serum ALT and AST activities. Following the treatment of MAFLD rats with various doses of WLS, a noticeable reduction in serum ALT and AST activities was observed. As shown in Figures 2(c) and 2(d), more lipid droplet vacuoles and ballooning were observed, and NAS was notably increased in the model group. The hepatic pathological damage and NAS were obviously improved after the treatment of WLS and PPC.

### 3.3. WLS Regulated Lipid Metabolism in MAFLD Rats

**Figure 3:**
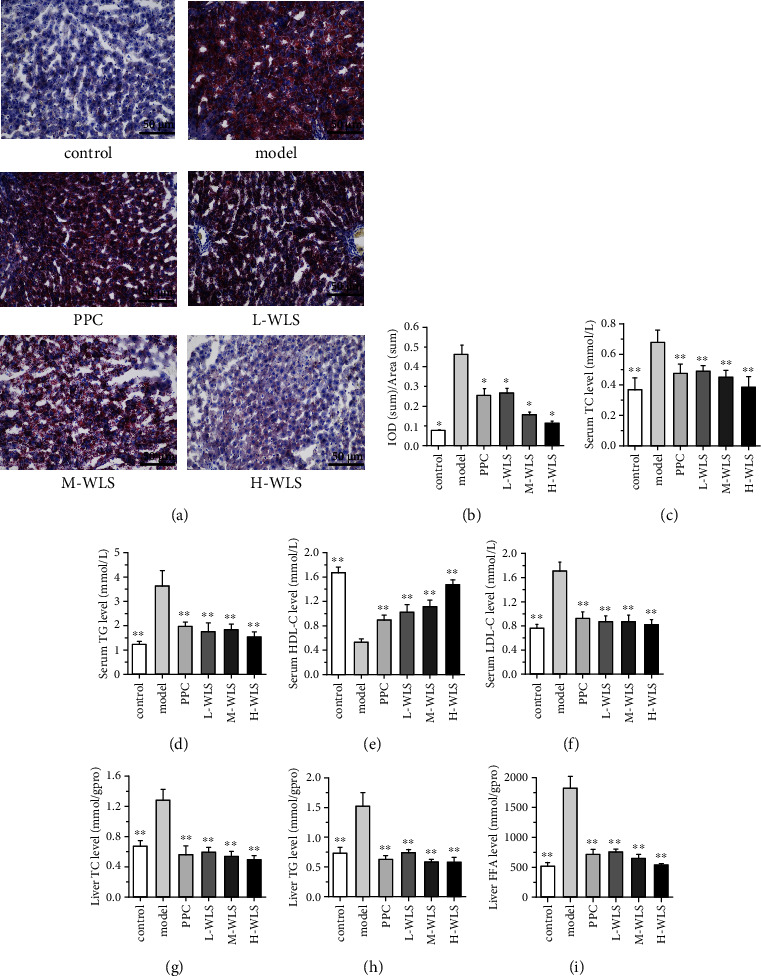
Effect of WLS on lipid metabolism in MAFLD rats. (a) Oil red O staining of liver tissue (×200). (b) Quantification of oil red O positive area. (c–f) The levels of TC, TG, LDL-C, and HDL-C in serum. (g–i) The levels of TC, TG, and FFA in the liver. Results were expressed as the mean ± SD, *n* = 3 in a–b, *n* = 10 in (c–i). ^∗^*P* < 0.05, ^∗∗^*P* < 0.01, compared with the model group.

Next, we employed oil red O staining to examine hepatic lipid deposition. As illustrated in Figures 3(a) and 3(b), the model group exhibited a significant increase in red lipid droplets compared to the control group. However, the WLS groups demonstrated a marked reduction in hepatic lipid accumulation.

The lipid levels in both serum and liver are presented in Figures 3(c)–3(i). In the model group, there was an elevation in serum lipids (TC, TG, and LDL-C) and hepatic lipids (TC, TG, and FFA). Conversely, the serum HDL-C level was decreased. However, treatment with WLS effectively reduced the levels of serum lipids (TC, TG, and LDL-C) and hepatic lipids (TC, TG, and FFA) while increasing serum HDL-C levels.

### 3.4. WLS Suppressed Oxidative Injury and Inflammation in MAFLD Rats

**Figure 4:**
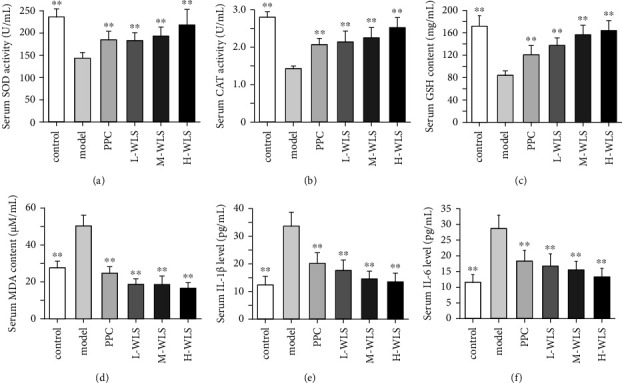
Effect of WLS on oxidative injury and inflammation in MAFLD rats. (a–f) The serum activities or contents of SOD, CAT, GSH, CAT, IL-1*β*, and IL-6. Results were expressed as the mean ± SD, *n* = 10. ^*∗∗*^*P* < 0.01, compared with the model group.

In the model group, there was the notable decrease in the activities or contents of SOD, CAT, and GSH in serum. Additionally, the levels of MDA, IL-1*β*, and IL-6 in serum were significantly increased. However, following 6 weeks of WLS treatment, there was a marked increase in the activities or contents of SOD, CAT, and GSH in serum, accompanied by a significant decrease in MDA, IL-1*β*, and IL-6 levels in serum (Figure 4).

### 3.5. WLS Ameliorated Lipid Deposition, Induced Autophagy, and the Activated AMPK/mTOR/ULK1 Signaling Pathway in MAFLD Rats

**Figure 5:**
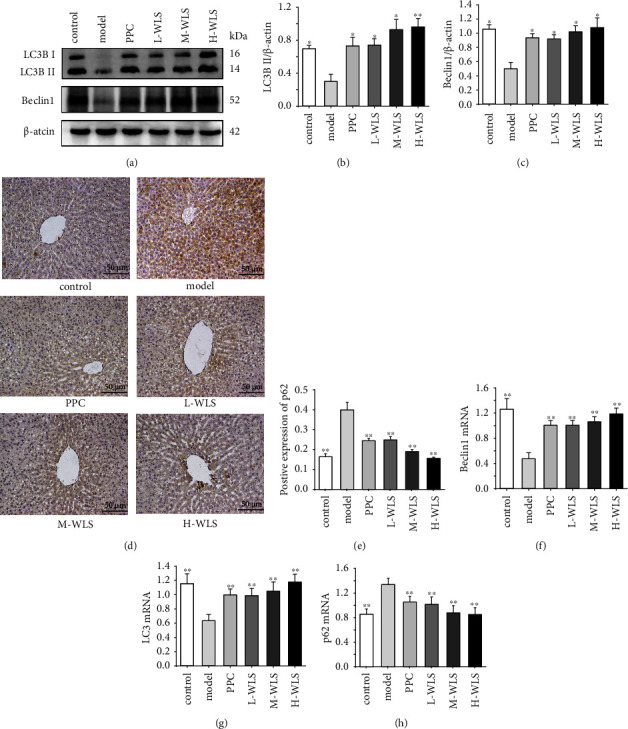
Effect of WLS on the proteins and genes of LC3B, Beclin1, and p62 in MAFLD rats. (a–e) Hepatic LC3B, Beclin1, and p62 proteins expressions. (f–h) Hepatic Beclin1, LC3B, and p62 genes expressions. Results were expressed as the mean ± SD, *n* = 3 in (a–e) and *n* = 6 in (f–h). ^*∗*^*P* < 0.05 and ^*∗∗*^*P* < 0.01, compared with the model group.

Subsequently, we assessed the levels of autophagy markers (Beclin1, LC3B, and p62) through western blot, qRT-PCR, and IHC to investigate the impact of WLS on autophagy in MAFLD rats (Figure 5). In the model group, the expressions of Beclin1 and LC3B-II were markedly downregulated, while p62 expression was significantly upregulated. However, following WLS treatment, there was a notable increase in the levels of Beclin1 and LC3B-II proteins, accompanied by a decrease in p62 expression.

**Figure 6:**
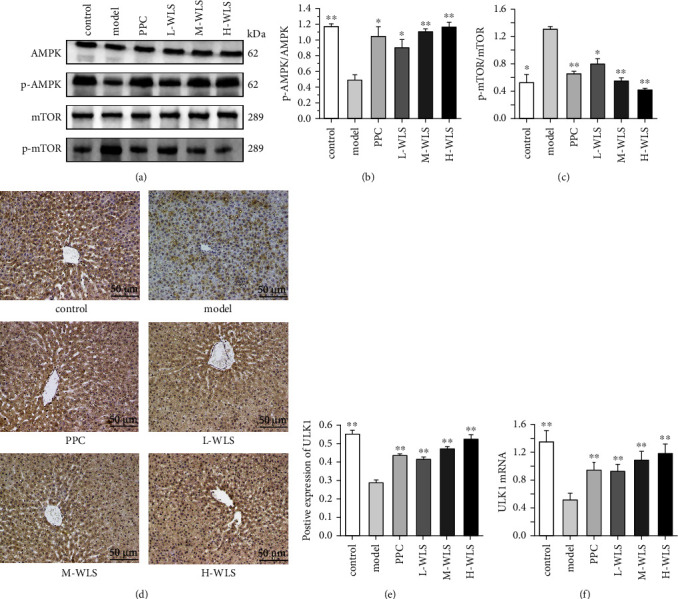
Effect of WLS on regulating the AMPK/mTOR/ULK1 signaling pathway in MAFLD rats. (a–c) Hepatic p-AMPK, p-mTOR proteins expressions. (d–f) Hepatic ULK1 protein and gene expression. Results were expressed as the mean ± SD, *n* = 3 in (a–e) and *n* = 6 in (f) ^*∗*^*P* < 0.05 and ^*∗∗*^*P* < 0.01, compared with the model group.

To ascertain whether WLS regulated the AMPK/mTOR/ULK1 signaling pathway to mediate autophagy, we evaluated the protein levels of AMPK, p-AMPK, mTOR, p-mTOR, and ULK1 in the liver. As depicted in Figure 6, the results demonstrated that the p-AMPK/AMPK and ULK1 expressions were decreased, whereas the p-mTOR/mTOR was elevated in the model group. However, WLS not only upregulated the p-AMPK/AMPK and ULK1 expressions but also downregulated the p-mTOR/mTOR. Additionally, qRT-PCR results indicated that WLS enhanced the expression of ULK1.

**Figure 7:**
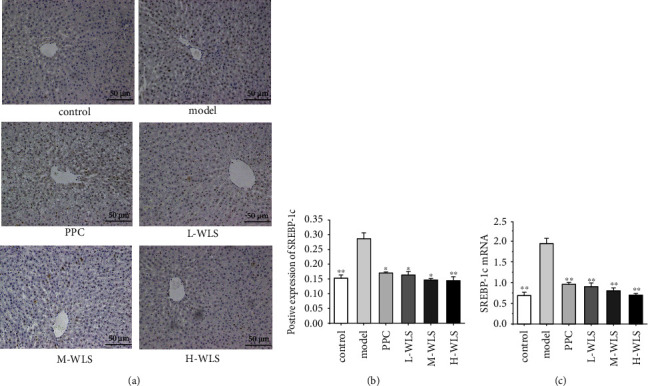
Effect of WLS on the expressions of SREBP-1c in MAFLD rats. (a–c) Expression of protein and gene of SREBP-1c in the liver. Results were expressed as the mean ± SD, *n* = 3 in (a, b) and *n* = 6 in (c). ^*∗*^*P* < 0.05 and ^*∗∗*^*P* < 0.01, compared with the model group.

Next, we examined the expression of SREBP-1c, a downstream factor of AMPK known to regulate lipid synthesis. Notably, WLS treatment led to a significant decrease in both the gene and protein expressions of SREBP-1c (Figure 7). Collectively, these findings indicate that WLS reduced lipid deposition and activated autophagy by modulating the AMPK/mTOR/ULK1 signaling pathway.

## 4. Discussion

In TCM, the spleen is responsible for transporting and transforming water and dampness. A sedentary lifestyle, irregular diet, or emotional disorders can lead to spleen dysfunction in transportation and the accumulation of dampness and phlegm, resulting in the occurrence of MAFLD. The formulation characteristics of WLS are consistent with the pathogenesis of spleen deficiency and phlegm-dampness in MAFLD. Zexie and zhuling could promote urination and drainage, fuling and baizhu have the effect of strengthening the spleen and removing dampness, and guizhi could warm the spleen *yang* and strengthen the spleen *qi*. Therefore, WLS may effectively strengthen the spleen, drain dampness, and eliminate phlegm. WLS has been used for thousands of years to treat renal disease [30, 31]. Clinically, WLS has been reported to significantly decrease lipid content [18].

Lipid metabolism disorder is the central link of the occurrence and progression of MAFLD. Modern medicine has found that dyslipidemia can be used as the microscopic manifestation of phlegm-dampness syndrome [32]. The serum levels of TC and TG in patients with phlegm-dampness syndrome were significantly higher than those in patients without phlegm-dampness syndrome, and the degree of hepatic lipid accumulation was directly proportional to the degree of phlegm dampness [33, 34]. Long-term HFD can cause severe liver injury, and the activities of ALT and AST in serum are the most sensitive indicators to evaluate the degree of liver injury. In our study, HFD increased the levels of serum lipids (TC, TG, and LDL-C) contents, hepatic lipids (TC, TC, FFA) contents, as well as the activities of ALT and AST in serum, and decreased the level of HDL-C in serum. These changes demonstrated that lipid metabolism disorder and liver injury occurred in the model group. Moreover, obvious hepatic steatosis in MAFLD rats was observed through HE and oil red O staining. Treatment with WLS notably decreased TC, TG, LDL-C, and FFA levels, as well as ALT and AST activities, while increasing the content of HDL-C. Histological analysis showed that WLS notably improved liver steatosis. Our results revealed the role of WLS in alleviating dyslipidemia and liver injury.

Autophagy is a cellular self-protection program activated during stress responses involving the recycling of harmful proteins and organelles through the lysosomal degradation pathway [35, 36]. Emerging evidence suggested that autophagy plays a role in regulating lipid metabolism through lysosome degradation [9]. Previous studies have demonstrated that inhibiting the expression of autophagy-associated proteins impairs the autophagy process, leading to the accumulation of lipid droplets and TG in liver, ultimately accelerating liver injury [37, 38]. Conversely, enhancing autophagy could improve mitochondrial function and mitigate the lipotoxicity caused by palmitic acid in mouse hepatocytes [39]. Therefore, promoting the activation of autophagy may serve as a potential strategy to regulate lipid metabolism in liver.

Autophagy is a continuous process, and Beclin1, p62, and LC3B are commonly used markers to assess autophagy dynamics [40]. Beclin1 is a key protein involved in regulating the initiation and nucleation of autophagosome formation, and its expression is positively correlated with autophagy [41]. Studies have shown that decreased Beclin1 expression disrupts autophagosome degradation in cultured cells [42]. LC3 consists of three isoforms: LC3A, LC3B, and LC3C, with LC3B being a highly conserved gene in mammals. During autophagosome formation, the plasma membrane-associated LC3B-I is conjugated with phosphatidylethanolamine at its C-terminus, forming LC3B-II [43]. Therefore, an increase in LC3B-II indicates autophagy activation and an increase in the number of autophagosomes. During autophagy, p62 binds to ubiquitinated proteins in the cytoplasm and subsequently interacts with LC3B-II on the autophagosome membrane. The complex is then degraded in autophagolysosomes [41]. Accumulation of p62 is a well-known characteristic of autophagy deficiency [44]. In our studies, we observed a decrease in LC3B-I to LC3B-II conversion and Beclin1 expression, as well as an upregulation of p62 in MAFLD rats. Conversely, treatment with WLS increased LC3B-II and Beclin1 expressions while decreasing p62 levels. These results indicated that WLS promotes autophagy in HFD-induced MAFLD rats.

AMPK/mTOR/ULK1-mediated autophagy activation has a positive effect on treating MAFLD. Autophagy is amplified by AMPK, a pivotal factor in maintaining energy homeostasis [45]. AMPK is a heterotrimeric protein consisting of *α*, *β*, and *γ* subunits. The regulatory subunits include *β* and *γ* subunits, while the catalytic subunit is the *α* subunit. The phosphorylation of threonine 172 residue on the *α* subunit is essential for AMPK activation [46]. mTOR can form TOR complex 1 (TORC1) and TOR complex 2 (TORC2) by interacting with various binding partners. The activation of mTORC1 suppresses autophagy [47, 48] Additionally, ULK1, an autophagy-initiating kinase, plays a pivotal role in autophagy initiation [49]. It is well established that AMPK and mTOR are essential for activating ULK1 to initiate autophagy [50]. On the one hand, mTORC1 phosphorylates Ser757 of ULK1, leading to the inhibition of its activity [17]. On the other hand, phosphorylated AMPK inactivates mTORC1 by phosphorylating tuberous sclerosis 2 and Raptor protein within mTORC1. Importantly, AMPK can directly activate ULK1 by phosphorylating Ser555, Ser317, and Ser777 of ULK1 [51]. SREBP-1c is mainly expressed in hepatocytes and adipocytes, and is a vital transcription factor that regulates lipogenesis by activating genes involved in fatty acid and triglyceride synthesis. Phosphorylated AMPK can phosphorylate the Ser372 residue of SREBP-1c to inhibit the expression of SREBP-1c, reducing fatty acid synthesis and lipid accumulation, and improving hepatic steatosis [52]. Our data demonstrated that WLS significantly phosphorylated AMPK and activated ULK1. Simultaneously, WLS treatment suppressed the activation of mTOR and SREBP-1c. These results collectively indicated that WLS alleviates HFD-induced MAFLD by regulating the AMPK/mTOR/ULK1 signaling pathway to promote autophagy and reduce lipid accumulation.

Meanwhile, the activation of autophagy also helps reduce oxidative stress and inflammatory responses. Autophagy plays a role in regulating the secretion of inflammatory cytokines. Studies have shown that the induction of autophagy pathways dependent on LC3B and p62 inhibits IL-1*β* and IL-6 production [53, 54]. IL-1*β* is mainly produced by hepatic macrophages and plays a major role in promoting insulin resistance, hepatic lipid accumulation, and inducing hepatic fibrosis [55]. IL-6 has chemotactic activities, which can promote local migration and infiltration of inflammatory cells into the fatty liver, thereby facilitating the cascade amplification of the inflammatory response in liver [56]. Further, autophagy mitigates oxidative stress by eliminating peroxides from damaged mitochondria and repairing damaged DNA [57, 58]. SOD, a metalloproteinase, can convert oxygen free radicals into hydrogen peroxide and molecular oxygen [59]. Hydrogen peroxide is harmful for body and could be immediately catalysed by CAT into completely harmless water [60]. GSH is a free radical scavenger, its content will lead to the accumulation of oxygen free radicals, increase the oxidative stress. Thus, the SOD, CAT and GSH activities indicates the capacity of the body's antioxidant. MDA as the production of lipid peroxidation, is regarded as oxidative stress of toxic molecules and biological markers. In our research, the administration of WLS significantly decreased the contents of IL-1*β*, IL-6, and MDA while increased SOD, GSH, and CAT activities and levels. These results demonstrated the antioxidant and anti-inflammatory effects of WLS. However, the mechanism of WLS activates autophagy to inhibit oxidative stress and inflammation reaction has not been clarified, which needs to be studied further.

## 5. Conclusion

In conclusion, our findings provided evidence that WLS can alleviate HFD-induced MAFLD by reducing lipid deposition and suppressing oxidative stress and inflammation through activating autophagy via regulating AMPK/mTOR/ULK1 signaling pathway. These results offered novel experimental evidence supporting the potential of targeting AMPK/mTOR/ULK1 signaling pathways as a therapeutic approach for MAFLD.

## Figures and Tables

**Table 1:** NAS scoring standard.

Histological marker	Score	Scoring standard
Steatosis	0	<5%
	1	5%–33%
	2	34%–66%
	3	>66%

Hepatocyte ballooning	0	None
	1	Few; inapparent
	2	Easily noted; many

Lobular inflammation	0	None
	1	<2
	2	2–4
	3	>4

**Table 2:** Primer sequence.

Gene	Forward	Reverse
Beclin1	F: AACTCTGGAGGTCTCGCTCT	R: CGCCTTAGACCCCTCCATTC
LC3B	F: TTGGTCAAGATCATCCGGCG	R: CGAAGGTTTCTTGGGAGGCA
p62	F: TGCTCCATCAGAGGATCCCA	R: TTTCTGCAGAGGTGGGTGTC
SREBP-1c	F: GTTAACGTGGGTCTCCTCCG	R: AGCATGTCTTCGATGTCGGT
ULK1	F: ACACACCCTCTCCCCAAGTG	R: GGTTCGTGGAGAGTGCTCAG
*β*-actin	F: TGCTATGTTGCCCTAGACTTCG	R: GTTGGCATAGAGGTCTTTACGG

**Table 3:** Detection wavelength, linear regression data, regression data, and content of 7 constituents in WLS.

Components	Λ (nm)	Content (mg/mg)	Regression equation	*r*
Ferulic acid	254	0.409 ± 0.010	*y* = 8*E* + 06*x* + 11381	0.991
Cinnamic acid	254	1.296 ± 0.016	*y* = 2*E* + 07*x* − 2239	1.000
Cinnamaldehyde	254	2.529 ± 0.019	*y* = 3*E* + 07*x* − 5075.7	1.000
Atractylenolide III	220	0.745 ± 0.006	*y* = 4*E* + 06*x* + 28.851	1.000
Alisol C-23-acetate	254	0.423 ± 0.005	*y* = 3*E* + 07*x* + 461.43	1.000
Atractylenolide II	220	0.110 ± 0.002	*y* = 4*E* + 07*x* + 9765.7	0.995
Atractylenolide I	254	0.120 ± 0.001	*y* = 4*E* + 07*x* − 10.045	1.000

## Data Availability

The data used to support the findings of this study are available from the corresponding author upon request.
